# Remission of insomnia in older adults treated with cognitive behavioral therapy for insomnia (CBT-I) reduces p16^INK4a^ gene expression in peripheral blood: secondary outcome analysis from a randomized clinical trial

**DOI:** 10.1007/s11357-023-00741-5

**Published:** 2023-02-28

**Authors:** Judith E. Carroll, Richard Olmstead, Steve W. Cole, Elizabeth C. Breen, Jesusa M. Arevalo, Michael R. Irwin

**Affiliations:** 1 Cousins Center for Psychoneuroimmunology, University of California, Los Angeles, Los Angeles, CA USA; 2 Jane & Terry Semel Institute for Neuroscience and Human Behavior, Department of Psychiatry & Biobehavioral Sciences, UCLA David Geffen School of Medicine, University of California, 300 UCLA Medical Plaza, Suite 3330, Los Angeles, CA 90095 USA

**Keywords:** Senescence, Insomnia, Intervention, Remission, Cognitive behavioral therapy, Aging

## Abstract

Late life insomnia may increase risk for accelerated biological aging. Intervening to treat insomnia may provide protection from biological aging by reducing the prevalence of senescent cells in the immune system, as indicated by gene expression of a marker of cellular senescence, p16^INK4a^. In the present study, we determine whether treatment of insomnia in older adults with cognitive behavioral therapy for insomnia (CBT-I) would reduce p16^INK4a^ gene expression in peripheral blood mononuclear cells (PBMC), compared to a sleep education therapy (SET), an active comparator condition. Secondly, we investigate the relationship between sustained insomnia remission and reduced expression of p16^INK4a^. Participants 60 + years old with insomnia were enrolled in a randomized controlled trial and assigned to CBT-I or SET. Analyses of 231 older adults (CBT-I = 119; SET = 112) examine baseline, post (2 months), and 24 months gene expression of p16^INK4a^. Compared to baseline, expression of p16^INK4a^ increased in the SET group over 24 months (*P* = 0.03), but showed no change in the CBT-I group. Those who received CBT-I and experienced sustained remission of insomnia had a significant decline in p16^INK4a^ expression by 24 months compared to baseline (*P* = 0.02). Individuals not sustaining remission of insomnia exhibited overall increase expression of p16^INK4a^ by 24 months (*P* = 0.03). In older adults with insomnia, p16^INK4a^ increases over 24 months, while CBT-I treatment of insomnia mitigates the increase in p16^INK4a^. Further, sustained remission of insomnia using CBT-I leads to a decrease in p16^INK4a^. These results suggest that behavioral interventions that are effective at treating insomnia might reduce the population of senescent cells in circulating blood.

**Supplementary Information:**

The online version contains supplementary material available at 10.1007/s11357-023-00741-5.

## Introduction

Insomnia is a disorder characterized by an inability to initiate or sustain sleep at night, accompanied by daytime dysfunction. Estimated prevalence of insomnia increases with age with roughly half of adults over age 65 reporting sleep disturbances, and 10–25% experiencing more severe clinical insomnia [1–4]. Insomnia among older adults raises risk for poorer health, frailty [5, 6], cognitive decline [7, 8], cardiovascular disease [9–11], cancer progression [12–14], and mortality [15, 16].

This effect of insomnia on health in older adults may be particularly detrimental in an aging biological system as aged systems have reduced capacity to respond to new insults. Aging within the immune system has implications for immune defenses, including reduced bacterial clearance, vaccine efficacy, defenses against viral attack, and slowed wound healing [17–21]. However, aging immune system can also have consequences for biological aging across tissues [17, 19, 22, 23], raising risk for chronic disease and death [24–26].

Although disease morbidity and mortality risk are elevated among those with sleep disturbances [1, 27–29], the specific molecular pathways altered by sleep loss, which impact human disease, are poorly defined, and these pathways may be particularly magnified in late life when there is reduced reserve capacity to rebound (i.e., resilience). Potential pathways include accelerating biological aging through an increase in cellular stress and accumulation of damage cells [30, 31] that enter a senescent state [32], which are a major contributor to biological aging [33–38].

Cellular senescence is a state of cell cycle arrest, commonly reached by cell replication (e.g., critically short telomeres) or excess cell stress (e.g., DNA damage) [17, 39–42]. Expression of p16(INK4a), a protein that inhibits cells from replicating, has been proposed as a biomarker of human aging, as it is expressed in cells that are senescent [43, 44]. Cellular senescence is thought to not only serve as a biomarker of aging but is also thought to promote the progression of biological aging, predominantly by increasing the release of proinflammatory and degrading secretory factors [41, 45–47]. Removal of senescent cells reverse or delay the aging pathology and improve lifespan in preclinical models [48, 49], resulting in the initiation of human trials to target these cells for elimination or reduction in diseases of aging [50], with the potential to impact cardiovascular disease progression, Alzheimer’s disease and related dementias, arthritis, osteoporosis, sarcopenia, and immunosenescence [51]. This burgeoning research that seeks to extend human *healthspan* by reducing and eliminating diseases of biological aging, coined Geroscience, has a need for the identification of modifiable behaviors that also may be directly targeted to slow the aging process [49, 52].

Insomnia is a modifiable behavioral target with established treatment efficacy that might alter biological aging trajectories. Cognitive behavioral therapy for insomnia (CBT-I) is an effective intervention to treat insomnia among older adults and typically includes cognitive therapy, stimulus control, sleep hygiene, and relaxation training [53, 54]. Insomnia is a behavior amendable to intervention but whether the successful remission of insomnia results in alterations to the expression of a marker of senescence has not been directly tested.

In the present study, we hypothesized that treatment of insomnia with CBT-I would reduce expression of p16^INK4a^ in peripheral blood mononuclear cells (PBMCs) compared to a sleep education therapy (SET), an active comparator condition. We further predicted that insomnia remission would protect from increases in p16^INK4a^, whereas those with sustained insomnia were expected to have increasing p16^INK4a^ gene expression.

## Methods

### Trial design and oversight

This trial was investigator initiated, single site, masked (rater), partially blinded (participants), parallel-group, randomized controlled trial with recruitment from July 1, 2012 through April 30, 2015. Full trial design has been reported previously [see Irwin et al., 2022 Protocol Supplement (Appendix)] [55]. The trial and the subsequent R01 to support the current aims was funded by National Institute of Aging, which had no influence on design or conduct of the trial, and was not involved in data analysis, interpretation, or manuscript writing. Approval was obtained from the institutional review board at the University of California, Los Angeles, and conducted in accordance with the provision of the Declaration of Helsinki and Good Clinical Practices guidelines. All participants gave written informed consent.

### Participants

Recruitment involved targeted enrollment of adults 60 years and older residing within 15 mi of the UCLA Westwood campus with current insomnia. Screening eligibility for enrollment assessed presence of general sleep disturbance, determined by self-report (i.e., 15-item Pittsburgh Sleep Quality Index, PSQI, score > 5) [56], and the absence of a current depression (i.e., 10-item Center for Epidemiologic Studies Depression [CESD] score < 4) [57]. Interviews confirmed that participants met criteria for insomnia using diagnostic criteria of the Diagnostic and Statistical Manual for Mental Disorders, fourth edition (DSM-IV), and absence of major depression using Structured Clinical Interview (SCID) for DSM-IV or DSM-V within the last 12 months. Full inclusion and exclusion criteria are provided in a prior published Protocol [55]. In brief, participants with major medical conditions such as cancer, recent stroke or myocardial infarction, neurological disease, autoimmune disorders, regular use of opioids, psychotropic, or steroids were excluded.

### Trial procedures

As reported previously [55], participants were randomized using a computer-generated random number sequence, with block sizes from 5 to 10 participants. Treatment allocation was masked so that research assessors were blinded to treatment allocation of the participants, *and participants were partially blinded*, i.e., *modified blind*-*to*-*treatment protocol*. CBT-I and SET interventions were delivered over an 8-week (2 months) period in weekly 120-min group sessions as reported previously. Briefly, CBT-I contained five validated components: cognitive therapy, stimulus control, sleep restriction, sleep hygiene, and relaxation. SET contained educational components: sleep hygiene, sleep biology, characteristics of healthy and unhealthy sleep, stress biology, and impact on sleep. SET is an active comparator as previously demonstrated [55], which was matched to CBT-I for time, attention, group interaction, and expectancy of benefit effects.

Peripheral blood samples were collected by venipuncture immediately prior to initiation of treatment (baseline), after completion of treatment (post, 2 months after baseline), and again at 24 months after baseline. PBMCs were isolated from heparinized whole blood by density gradient centrifugation within 2 h of blood draw. Up to 5 × 10^6^ PBMCs were resuspended in RLT Lysis Buffer (QIAGEN) plus β–2-mercaptoethanol for the preservation of mRNA, and frozen at – 80 °C until the completion of the study.

#### Insomnia remission

Remission of insomnia was determined using SCID DSM-IV criterion for insomnia at each post intervention visit as described previously [55]. Percentage of visits with no insomnia was calculated and used to determine two categories: (1) sustained remission (100% of follow up assessments with no insomnia) and (2) no sustained remission of insomnia (any follow up visits with insomnia).

### Outcome

The primary outcome was the relative abundance of mRNA transcripts from the *CDKN2A* gene, which encodes p16^INK4a^, a biomarker for cellular senescence and aging, in peripheral blood mononuclear cells (PBMCs). Assays were conducted as previously described [58], with preserved and frozen PBMC samples thawed and total RNA extracted (QIAGEN RNeasy), tested for suitable mass (PicoGreen RNA) and integrity (Agilent TapeStation), reverse-transcribed to cDNA (Illumina TruSeq stranded), and sequenced on an Illumina HiSeq 4000 instrument using the manufacturer’s standard protocols. Assays targeted 10 million reads per sample (achieved average = 14.4 million), each of which was mapped to the GRCh38 reference human transcriptome using the STAR aligner, with *CDKN2A* mRNA abundance quantified as transcripts per million total mapped reads. Transcript abundance data were log2 transformed for analysis as described below.

### Statistical analyses

Analyses are aligned with CONSORT guidelines and were defined in a statistical analysis plan consistent with the primary trial results.^57^ IBM SPSS Version 27 was used for all analyses. Characteristics of participants at baseline enrollment are reported and compared by treatment group (CBT-I vs. SET) using Wilcox and chi-square tests for differences. Primary outcome analyses tested *hypothesis 1*: relative to SET, CBT-I will have lower overall expression of p16^INK4a^ over time; *hypothesis 2*: relative to unremitting insomnia, remission will be associated with less overall expression of p16^INK4a^ over time, and this will be most pronounced in the CBT-I group. These hypotheses were analyzed using linear mixed models, including all subjects in the intent-to-treat cohort. To test hypothesis 1, a 2 (CBT-I vs. SET) by 3 (baseline, post, 24 months) analysis was performed. Hypothesis 2 was tested using a 4 (CBT-I with remission, CBT-I without remission, SET with remission, SET without remission) by 3 (baseline, post, 24 months) design. Following this, mean differences between groups were evaluated using Fisher’s least significant difference contrast. The primary model (model 0) does not control for covariates, providing an unconditional (generalizable) estimate of treatment effects on p16^INK4a^. It was anticipated that randomization procedures would generate group equivalents on demographic, psychosocial, biobehavioral, and medical factors (i.e., age, BMI, gender, education, comorbidities, etc.); however, potential confounding variables, plus gene expression at baseline, were considered in a subsequent model (model 1) to determine whether the observed effects hold up when these variables are controlled. Given that depression was previously reported to be higher in the SET group compared to CBT-I group [55], we conducted additional secondary analyses controlling for incident depression in the model. While the sample size target was determined by the primary aims of the main trial, the present sample size provides greater than 80% power to detect modest differences (*d* ≥ 0.35) within secondary analyses. “Minimally important differences” for health outcomes have been suggested to fall within the range of *d* =  − 0.30 to *d* = 0.50 [59, 60].

## Results

### Participants and treatment

**Fig. 1:**
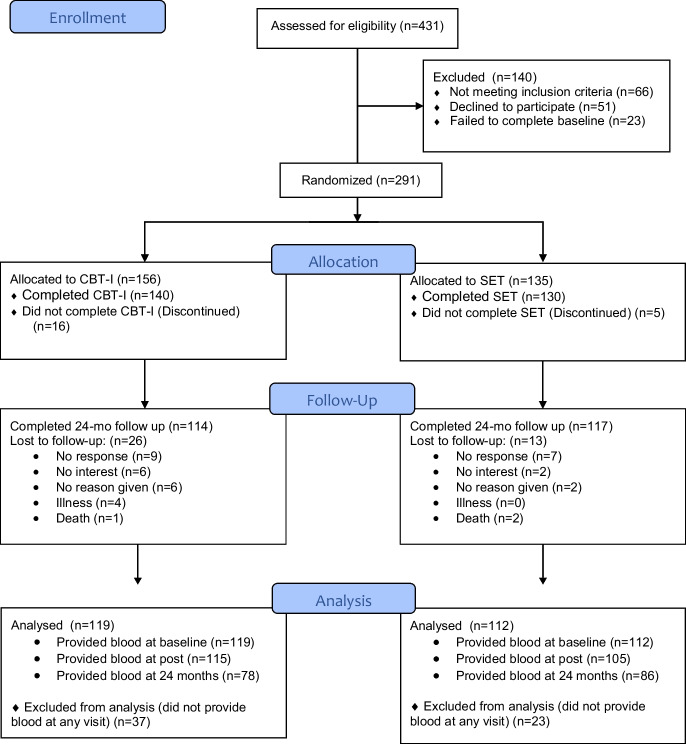
CONSORT flow diagram

A total of 291 participants were enrolled in the primary trial from August 2012 to April 2015, with 156 assigned to CBT-I and 135 to the SET group. Of the enrolled participants, a total of 231 (119 CBT-I; 112 SET) provided blood specimens at baseline plus at least one of two subsequent follow up visits to ascertain our primary outcome, p16^INK4a^ (see Fig. 1, CONSORT). Characteristics of participants enrolled in the trial who did not provide blood did not differ from those providing blood except for a modest difference in gender (data in supplement Table 1). Among participants in the current analysis, those enrolled in the CBT-I compared to those in the SET group had on average a higher BMI and a higher number of years of education (Table 1). No other demographic or clinical feature differed between groups.

**Table 1:** Baseline sample characteristics by treatment group

	CBT-I (*n* = 119)	SET (*n* = 112)		
	Mean (SD) or %	Mean (SD) or %	*t* (*x*^2^)	*P*
Age (years)	70.2 (7.0)	69.7 (6.0)	0.65	0.52
Race (% White)	84.6%	83.9%	0.02	0.89
Gender (% Female)	51.3%	58.0%	1.07	0.30
Body Mass Index (BMI, kg/m^2^)	27.4 (4.2)	26.0 (4.1)	2.51	0.013
Education (Years)	17.1 (2.5)	16.4 (2.4)	2.23	0.027
Smoker (% Yes)	2.5%	6.3%	1.98	0.16
Comorbidity Score	2.8 (1.1)	2.7 (0.8)	0.39	0.70
Baseline log2 p16^INK4a^ gene expression	0.68 (1.6)	0.65 (1.6)	0.15	0.88

Comorbidity score is derived from the Charlson Comorbidity Index [72]

Treatment adherence and acceptability did not differ between treatment arms, as reported previously [55]. For the primary outcome data, p16^INK4a^, the pattern of missing data was not different across groups (X^2^ = 2.11; *P* = 0.15).

### Primary outcome

**Fig. 2:**
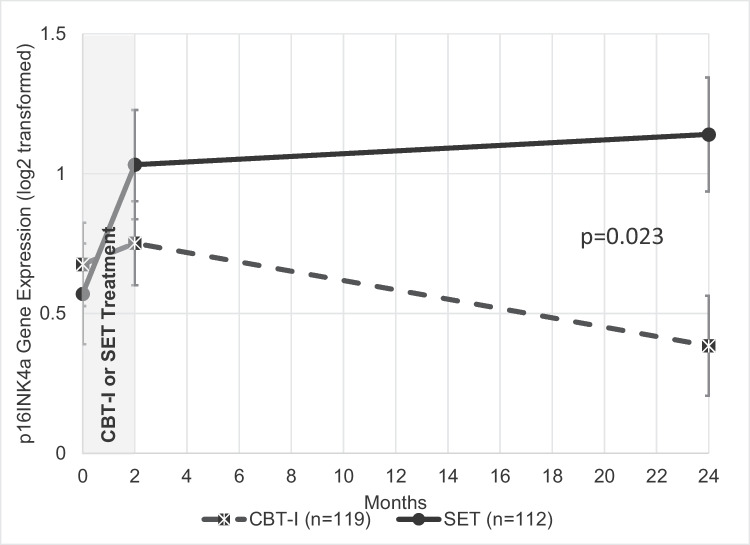
The effect of treatment on p16^INK4a^ gene expression. The adjusted means and standard errors for log2 transformed p16INK4a are derived from mixed linear models including all subjects in intent-to-treat analyses, and graphically displayed in the figure for the two treatment groups, cognitive behavioral therapy for insomnia (CBT-I) (dashed line) and sleep education therapy (SET) (solid line) at baseline (prior to treatment), following treatment (2 months), and 24 months after treatment initiation. The *p* value shown is for the time by treatment interaction

**Fig. 3:**
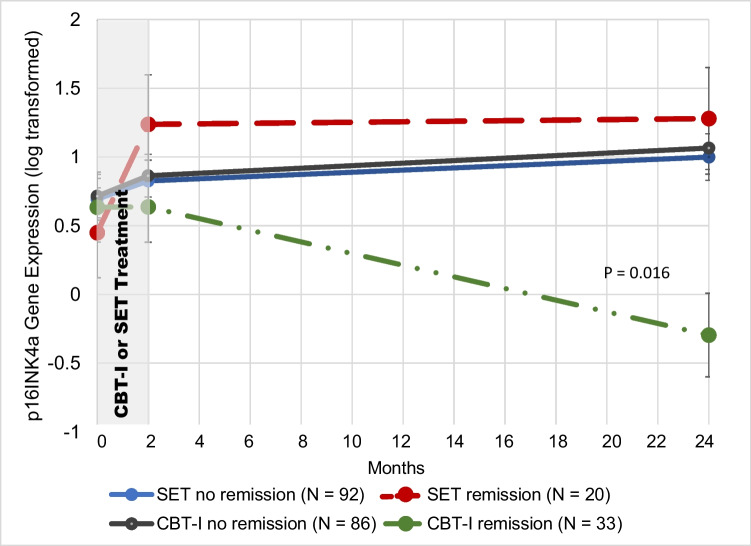
The effect of treatment and sustained remission status on p16^INK4a^ gene expression. The adjusted means and standard errors for log2 transformed p16INK4a are derived from mixed linear models including all subjects in intent-to-treat analyses, and graphically displayed in the figure for baseline (prior to treatment), following treatment (2 months), and 24 months after treatment initiation for four groups, sleep education treatment (SET) without remission (Blue), SET with sustained remission (red), cognitive behavioral therapy for insomnia (CBT-I) without remission (Black), and CBT-I with sustained insomnia remission (green line). The *P* value shown is for the group by time interaction

Analyses of the effect of treatment condition (CBT-I vs SET) on p16^INK4a^ expression over time (baseline, post, 24 months) was significant (*P* = 0.023), such that participants assigned to the SET condition had increased expression of p16^INK4a^ over 24 months, while those assigned to the CBT-I exhibited no increases in p16^INK4a^ expression over 24 months (Fig. 2; Table 2). The proportion of participants who achieved remission of insomnia disorder immediately following treatment (2 months after baseline) in the current sample was greater in the CBT-I group (61 [51.3%]) compared to the SET group (41 [37.3%], adjusted β = 0.67; 95%CI, 0.12–0.1.00; *P* = 0.02). For sustained remission of insomnia disorder (i.e., insomnia remission 24 months after baseline), similar results were found in the CBT-I group (33 [27.7%]) compared with the SET group (20 [17.9%]; adjusted β = 0.77; 95% CI, 0.10–1.00; *P* = 0.03). Linear mixed models testing the interaction of treatment with and without remission (4 groups) on p16^INK4a^ over time (3 timepoints) was significant (*P* = 0.016; Table 2; Fig. 3). Insomnia remission in the CBT-I group was associated with the greatest difference in p16INK4a expression values over time, such that those enrolled in the CBT-I who had full remission of insomnia exhibited a significant decrease in p16^INK4a^ by 24 months, a significant change from baseline in this group (*P* = 0.02). Individuals not sustaining remission of insomnia in the CBT-I or SET group exhibited overall increase expression of p16^INK4a^ by 24 months (*P* = 0.025).

**Table 2:** Mixed model effect of treatment group and remission status on gene expression of p16^INK4a^ (*N* = 231 total participants, all available timepoints)

	Model 0		Model 1, Adjusting for age, BMI, education, comorbidity, baseline gene expression	
	*F* (*df*)	*P*	*F* (*df*)	*P*
*p16*^*INK4a*^* gene expression*				
Time^a^	1.73 (2, 398.0)	0.18	1.20 (2, 407.7)	0.31
Treatment	3.08 (1, 228.7)	0.081	14.16 (1, 197.4)	< 0.001
Remission	1.31 (1, 228.7)	0.26	0.84 (1, 201.5)	0.37
Treatment × time	3.82 (2, 398.0)	0.023	3.06 (2, 409.2)	0.048
Time^a^	1.72 (2, 398.0)	0.18	1.20 (2, 407.7)	0.31
Four group^b^	2.42 (3, 228.5)	0.067	6.46 (3, 198.9)	< 0.001
Time × four groups	2.64 (6, 396.3)	0.016	2.08 (6, 406.2)	0.055

^a^Three timepoints: baseline, post (2 months), 24 months

^b^Four groups: cognitive behavioral therapy for insomnia (CBT-I) with remission, CBT-I without remission, sleep education therapy (SET) with remission, SET without remission

### Secondary analyses

Secondary analyses further adjusted for age, BMI, gender, education, and comorbidities, plus the baseline level of p16^INK4a^ expression. The effect of treatment condition on p16^INK4a^ expression over time remained significant (*P* = 0.048), and analyses testing insomnia remission in the CBT-I group were similar after adjustment for covariates (*P* = 0.055) (Table 2). Further adjusting for depression incidence had no effect on the results in Table 2 (data not shown).

## Discussion

The current study examined whether a randomized clinical trial to treat insomnia among older adults could result in modification of a hallmark of biological aging, namely cellular senescence. Older adults assigned to a cognitive behavioral therapy for insomnia had no increases over 2 years in the expression of the gene for p16^INK4a^, a marker of cellular senescence, in peripheral blood mononuclear cells. In contrast, those assigned to the control arm of the study had significant increases in p16^INK4a^ gene expression over the same 2-year time interval. In analyses of individuals with sustained insomnia remission 2 years after the initiation of cognitive behavioral therapy treatment, we observed significant decreases in cellular senescence-associated gene expression over 2 years, while those with chronic unremitting insomnia had increased cell senescence gene expression over 2 years. These results point to a role of insomnia in accelerating biological aging, while successful treatment and remission of insomnia resulted in reduction in this aging marker.

One in four older adults suffer from moderate to severe clinical insomnia [1–4], making this a highly salient modifiable behavioral factor that, when treated successfully, may slow the aging process by reducing cell senescence. As insomnia raises risk for poorer health, frailty [5, 6], cognitive decline [7, 8], cardiovascular disease [9–11], cancer progression [12–14], and mortality [15, 16], there is a high need to address this modifiable target in clinical practice. Our prior work identified lowered inflammation and reduced risk profiles for disease following successful remission of insomnia in older adults receiving either CBT-I or Tai Chi interventions [61, 62]. This study extends these results, suggesting that successful remission of insomnia alters levels of a marker of cellular senescence in circulating blood. Future research will need to better understand how insomnia drives aging and the mechanisms that are driving declines in p16^INK4a^ expression levels following successful remission. Long-term follow-ups of individuals with successful remission of insomnia compared to those with ongoing chronic insomnia would be needed to understand the lasting effect of the treatment on biological aging and risk for disease.

Another feature of this study that may have strengthened the results is the characteristics of the cohort. As aged systems are thought to be less resilient to challenges such as sleep loss, our intervention may have a stronger effect in an older adult sample than if the intervention was delivered to younger adults with insomnia. Additional research will be necessary to test vulnerability and resilience to the efficacy of the intervention to slow biological aging across the lifespan.

Of note, our marker of cellular senescence captures aging within the circulating immune system, which has implications for immunity [17–21], but may also contribute to aging processes across other tissues in the body [17, 19, 22, 23], and be a link to chronic disease and death [24–26]. Specific molecular pathways altered by insomnia and treatment of insomnia are not entirely clear. Similar to studies testing the biological pathways driving aging in response to chronic stress exposure [63–65], insomnia is associated with elevated anxiety, stress responsivity, and activated catecholaminergic system [66]. Activation of this system is thought to drive inflammation [67] and elevate mitochondrial metabolism, raising cellular stress and accumulation of damage [30, 31, 64]. These pathways are involved in driving aging and cellular senescence [48]. In contrast, a reduction in the overall expression of a senescence-related gene following improvements in sleep may indicate enhanced clearance of senescent cells during sleep intervals, parallel to research investigating the importance of sleep for clearing waste products in the brain [68, 69], or may reflect reduced development of expression (and therefore, senescence) within the PBMC population with improved sleep, resulting in less overall gene expression over time. Testing the pathways specifically involved in altering the expression of p16^INK4a^ in PBMCs following remission of insomnia by CBT-I will be an important next step.

Despite many strengths of the current study design including a randomized clinical trial, detailed assessments of insomnia status and covariates, and a longitudinal follow up of 2 years, a few limitations of the current study should be noted. First, our sample was comprised of moderately mobile and healthy older adults and may not generalize to those with multimorbidity, frailty, or chronic inflammatory conditions. Second, as our sample was comprised of older adults, the results need to be replicated in other age groups. Last, the sample was representative of the nearby community of patients at our institution, predominantly Caucasian and of upper middle income, and results of the trial may differ when applying it to other socioeconomic or ethnic/racial groups with divergent risk. Further research is needed to understand the role of insomnia remission at slowing biological aging in populations of different racial and ethnic make-up, ages, regions of the US and other countries, and economic backgrounds.

## Conclusions

The current randomized clinical trial of Cognitive Behavioral Therapy for Insomnia (CBT-I) compared to a sleep education control group was effective at altering the gene expression for the biological aging biomarker p16^INK4a^ in older adult patients initially diagnosed with insomnia. Those in the CBT-I group who experienced sustained remission of insomnia had significant lowering of p16^INK4a^ gene expression while those in the control group who had sustained insomnia over the 2-year interval exhibited increases in p16INK4a. These results extend prior work linking insomnia in late life with hallmarks of biological aging [70, 71], and point to the utility of an effective intervention to treat insomnia [53, 54] which may also be useful for the field of Geroscience by identifying a modifiable behavior (i.e., insomnia) that alters one key element of the aging process, cellular senescence.

### Supplementary Information

Below is the link to the electronic supplementary material.

Supplementary file1 (DOCX 15 kb)

## References

[1] Ohayon MM (2002). Epidemiology of insomnia: what we know and what we still need to learn. Sleep Med Rev.

[2] Morin CM, LeBlanc M, Daley M, Gregoire JP, Mérette C (2006). Epidemiology of insomnia: prevalence, self-help treatments, consultations, and determinants of help-seeking behaviors. Sleep Med.

[3] Leger D, Guilleminault C, Dreyfus JP, Delahaye C, Paillard M (2000). Prevalence of insomnia in a survey of 12 778 adults in France. J Sleep Res.

[4] Dzierzewski JM, O’Brien EM, Kay D, McCrae CS (2010). Tackling sleeplessness: psychological treatment options for insomnia in older adults. Nat Sci Sleep.

[5] Ensrud KE, Blackwell TL, Ancoli-Israel S (2012). Sleep disturbances and risk of frailty and mortality in older men. Sleep Med.

[6] Del Brutto OH, Mera RM, Sedler MJ (2016). The effect of age in the association between frailty and poor sleep quality: a population-based study in community-dwellers (The Atahualpa Project). J Am Med Dir Assoc.

[7] Tsapanou A, Vlachos GS, Cosentino S (2019). Sleep and subjective cognitive decline in cognitively healthy elderly: Results from two cohorts. J Sleep Res..

[8] Cricco M, Simonsick EM, Foley DJ (2001). The impact of insomnia on cognitive functioning in older adults. J Am Geriatr Soc.

[9] Jackson CL, Redline S, Emmons KM (2015). Sleep as a potential fundamental contributor to disparities in cardiovascular health. Annu Rev Public Health.

[10] Irwin MR (2015). Why sleep is important for health: a psychoneuroimmunology perspective. Annu Rev Psychol..

[11] Sands-Lincoln M, Loucks EB, Lu B (2013). Sleep duration, insomnia, and coronary heart disease among postmenopausal women in the Women’s Health Initiative. J Womens Health (Larchmt).

[12] Savard J, Morin CM (2001). Insomnia in the context of cancer: a review of a neglected problem. J Clin Oncol.

[13] Santoso AMM, Jansen F, de Vries R, Leemans CR, van Straten A, Verdonck-de Leeuw IM (2019). Prevalence of sleep disturbances among head and neck cancer patients: A systematic review and meta-analysis. Sleep Med Rev.

[14] Savard J, Ivers H, Villa J, Caplette-Gingras A, Morin CM (2011). Natural course of insomnia comorbid with cancer: an 18-month longitudinal study. J Clin Oncol.

[15] Althuis MD, Fredman L, Langenberg PW, Magaziner J (1998). The relationship between insomnia and mortality among community-dwelling older women. J Am Geriatr Soc.

[16] Kripke DF (2002). Mortality associated with sleep duration and insomnia. Arch Gen Psychiatry.

[17] Effros RB (2005). The role of CD8 T cell replicative senescence in human aging. Discov Med.

[18] Weinberger B, Herndler-Brandstetter D, Schwanninger A, Weiskopf D, Grubeck-Loebenstein B (2008). Biology of immune responses to vaccines in elderly persons. Clin Infect Dis.

[19] Ovadya Y, Landsberger T, Leins H (2018). Impaired immune surveillance accelerates accumulation of senescent cells and aging. Nat Commun.

[20] Linton PJ, Dorshkind K (2004). Age-related changes in lymphocyte development and function. Nat Immunol.

[21] Dorshkind K, Montecino-Rodriguez E, Signer RAJ (2009). The ageing immune system: is it ever too old to become young again?. Nat Rev Immunol.

[22] Yousefzadeh MJ, Flores RR, Zhu Y (2021). An aged immune system drives senescence and ageing of solid organs. Nature.

[23] Chou JP, Effros RB (2013). T cell replicative senescence in human aging. Curr Pharm Des.

[24] Sahin E, Colla S, Liesa M (2011). Telomere dysfunction induces metabolic and mitochondrial compromise. Nature.

[25] Taffett GE. Physiology of Aging. In: Cassel CK, ed. *Geriatric medicine: an evidence-based approach*. 4th ed. Springer Science & Business Media; 2003:27–35.

[26] Armbrecht JH, Sinclair AJ, Morley JE, Vellas B (2012). A biological perspective of ageing. Pathy’s Principles and Practice of Geriatric Medicine.

[27] Center for Disease Control. CDC Data & Statistics | Feature: insufficient sleep is a public health epidemic. Published 2011. Accessed August 4, 2011. http://www.cdc.gov/Features/dsSleep/.

[28] Colten HR, Altevogt BM (eds.), Institute of Medicine (US) Committee on Sleep Medicine and Research. Sleep disorders and sleep deprivation: An unmet public health problem. Washington, DC: National Academies Press; 2006. 20669438

[29] Motivala SJ (2011). Sleep and inflammation: psychoneuroimmunology in the context of cardiovascular disease. Ann Behav Med.

[30] Carroll JE, Cole SW, Seeman TE (2016). Partial sleep deprivation activates the DNA damage response (DDR) and the senescence-associated secretory phenotype (SASP) in aged adult humans. Brain Behav Immun.

[31] Carroll JE, Prather AA (2021). Sleep and biological aging: a short review. Curr Opin Endocr Metab Res.

[32] Everson CA, Henchen CJ, Szabo A, Hogg N (2014). Cell injury and repair resulting from sleep loss and sleep recovery in laboratory rats. Sleep.

[33] Cribbet MR, Carlisle M, Cawthon RM (2014). Cellular aging and restorative processes: subjective sleep quality and duration moderate the association between age and telomere length in a sample of middle-aged and older adults. Sleep.

[34] Jackowska M, Hamer M, Carvalho LA, Erusalimsky JD, Butcher L, Steptoe A (2012). Short sleep duration is associated with shorter telomere length in healthy men: findings from the Whitehall II cohort study. Kiechl S, ed. PLoS One..

[35] Prather AA, Puterman E, Lin J (2011). Shorter leukocyte telomere length in midlife women with poor sleep quality. J Aging Res.

[36] Liang G, Schernhammer E, Qi L, Gao X, De Vivo I, Han J (2011). Associations between Rotating Night Shifts, Sleep Duration, and Telomere Length in Women. Vina J, ed. PLoS One..

[37] Chen S, Lin J, Matsuguchi T (2014). Short leukocyte telomere length predicts incidence and progression of carotid atherosclerosis in American Indians: The Strong Heart Family Study. Aging (Albany NY).

[38] Prather AA, Gurfein B, Moran P (2015). Tired telomeres: poor global sleep quality, perceived stress, and telomere length in immune cell subsets in obese men and women. Brain Behav Immun.

[39] Blackburn EH (2005). Telomeres and telomerase: their mechanisms of action and the effects of altering their functions. FEBS Lett.

[40] Blackburn EH (2000). Telomere states and cell fates. Nature.

[41] Campisi J, d’Adda di Fagagna F (2007). Cellular senescence: when bad things happen to good cells. Nat Rev Mol Cell Biol..

[42] Campisi J (2005). Senescent cells, tumor suppression, and organismal aging: good citizens, bad neighbors. Cell.

[43] Liu Y, Sanoff HK, Cho H (2009). Expression of p16(INK4a) in peripheral blood T-cells is a biomarker of human aging. Aging Cell.

[44] Krishnamurthy J, Torrice C, Ramsey MR (2004). Ink4a/Arf expression is a biomarker of aging. J Clin Invest.

[45] Coppé J-P, Patil CK, Rodier F (2008). Senescence-associated secretory phenotypes reveal cell-nonautonomous functions of oncogenic RAS and the p53 tumor suppressor. PLoS Biol.

[46] Freund A, Orjalo AV, Desprez P-Y, Campisi J (2010). Inflammatory networks during cellular senescence: causes and consequences. Trends Mol Med.

[47] Coppé J-P, Desprez P-Y, Krtolica A, Campisi J (2010). The senescence-associated secretory phenotype: the dark side of tumor suppression. Annu Rev Pathol.

[48] López-Otín C, Blasco MA, Partridge L, Serrano M, Kroemer G (2013). The hallmarks of aging. Cell.

[49] Kennedy BK, Berger SL, Brunet A (2014). Geroscience: Linking Aging to Chronic Disease. Cell.

[50] Kirkland JL, Tchkonia T, Zhu Y, Niedernhofer LJ, Robbins PD (2017). The clinical potential of senolytic drugs. J Am Geriatr Soc.

[51] Collins F. Connecting Senescent Cells to Obesity and Anxiety. *NIH Director’s Blog*. January 8, 2019.

[52] Justice JN, Ferrucci L, Newman AB (2018). A framework for selection of blood-based biomarkers for geroscience-guided clinical trials: report from the TAME Biomarkers Workgroup. GeroScience.

[53] Irwin MR, Olmstead R, Carrillo C, et al. Tai Chi Chih compared with cognitive behavioral therapy for the treatment of insomnia in survivors of breast cancer: a randomized, partially blinded, noninferiority trial. J Clin Oncol. Published online May 10, 2017:JCO.2016.71.028. 10.1200/JCO.2016.71.0285. 10.1200/JCO.2016.71.0285 PMC5549450 28489508

[54] Morin CM, Bootzin RR, Buysse DJ, Edinger JD, Espie CA, Lichstein KL (2006). Psychological and behavioral treatment of insomnia: update of the recent evidence (1998–2004). Sleep.

[55] Irwin MR, Carrillo C, Sadeghi N, Bjurstrom MF, Breen EC, Olmstead R (2022). Prevention of incident and recurrent major depression in older adults with insomnia: a randomized clinical trial. JAMA Psychiat.

[56] Buysse DJ, Reynolds CF, Monk TH, Berman SR, Kupfer DJ (1989). The Pittsburgh Sleep Quality Index: a new instrument for psychiatric practice and research. Psychiatry Res.

[57] Roberts RE, Vernon SW (1983). The Center for Epidemiological Studies Depression Scale: Its use in a community sample. Am J Psychiatry.

[58] Cole SW, Shanahan MJ, Gaydosh L, Harris KM (2020). Population-based RNA profiling in Add Health finds social disparities in inflammatory and antiviral gene regulation to emerge by young adulthood. Proc Natl Acad Sci U S A.

[59] Hays RD, Farivar SS, Liu H (2005). Approaches and recommendations for estimating minimally important differences for health-related quality of life measures. COPD.

[60] Farivar SS, Liu H, Hays RD (2004). Half standard deviation estimate of the minimally important difference in HRQOL scores?. Expert Rev Pharmacoecon Outcomes Res.

[61] Carroll JE, Seeman TE, Olmstead R (2015). Improved sleep quality in older adults with insomnia reduces biomarkers of disease risk: Pilot results from a randomized controlled comparative efficacy trial. Psychoneuroendocrinology.

[62] Irwin MR, Olmstead R, Carrillo C (2014). Cognitive behavioral therapy vs. Tai Chi for late life insomnia and inflammatory risk: a randomized controlled comparative efficacy trial. Sleep..

[63] Rentscher KE, Carroll JE, Repetti RL, Cole SW, Reynolds BM, Robles TF. Chronic stress exposure and daily stress appraisals relate to biological aging marker p16 ^INK4a^. *Psychoneuroendocrinology*. 2019;102. 10.1016/j.psyneuen.2018.12.006. 10.1016/j.psyneuen.2018.12.006 PMC6420375 30557761

[64] Polsky LR, Rentscher KE, Carroll JE (2022). Stress-induced biological aging: A review and guide for research priorities. Brain Behav Immun.

[65] Prather AA, Epel ES, Portela Parra E (2018). Associations between chronic caregiving stress and T cell markers implicated in immunosenescence. Brain Behav Immun.

[66] Irwin M, Clark C, Kennedy B, Christian Gillin J, Ziegler M (2003). Nocturnal catecholamines and immune function in insomniacs, depressed patients, and control subjects. Brain Behav Immun.

[67] Irwin MR, Cole SW (2011). Reciprocal regulation of the neural and innate immune systems. Nat Rev Immunol.

[68] Iliff JJ, Lee H, Yu M (2013). Brain-wide pathway for waste clearance captured by contrast-enhanced MRI. J Clin Invest.

[69] Nedergaard M, Goldman SA (2020). Glymphatic failure as a final common pathway to dementia. Science (80-).

[70] Carroll JE, Esquivel S, Goldberg A (2016). Insomnia and telomere length in older adults. Sleep.

[71] Carroll JE, Irwin MR, Levine M (2017). Epigenetic aging and immune senescence in women with insomnia symptoms: findings from the Women’s Health Initiative Study. Biol Psychiatry.

[72] Charlson ME, Pompei P, Ales KL, MacKenzie CR (1987). A new method of classifying prognostic comorbidity in longitudinal studies: development and validation. J Chronic Dis.

