# Supplementary material for: Remission of insomnia in older adults treated with cognitive behavioral therapy for insomnia (CBT-I) reduces p16INK4a gene expression in peripheral blood: secondary outcome analysis from a randomized clinical trial
Source: GeroScience. 2023 Feb 28;45(4):2325–35. doi: 10.1007/s11357-023-00741-5 (PMC10651570; doi:10.1007/s11357-023-00741-5)
Supplement: Supplementary file 1 — Supplementary file1 (DOCX 15 kb) [file 11357_2023_741_MOESM1_ESM.docx]

| **Supplemental Table 1. Baseline Sample Characteristics of enrolled participants who provided blood compared to those who did not provide blood.** | | | | | | |
| --- | --- | --- | --- | --- | --- | --- |
|  | Provided blood  (n = 231) | | Did not provide blood  (n = 60) | |  | |
|  | *Mean (SD)* |  | *Mean (SD)* |  | *t* | *p* |
|  | *%* |  | *%* |  | *(x^2^)* |  |
|  |  |  |  |  |  |  |
| Age (years) | 70.0 (6.5) |  | 70.5 (7.5) |  | 0.59 | 0.56 |
|  |  |  |  |  |  |  |
| Race (% White) | 84.3% |  | 80.0% |  | 0.61 | 0.44 |
|  |  |  |  |  |  |  |
| Gender (% Female) | 54.5% |  | 70.0% |  | 4.80 | 0.03 |
|  |  |  |  |  |  |  |
| Body Mass Index (BMI, kg/m^2^) | 26.7 (4.2) |  | 26.8 (5.0) |  | 0.10 | 0.92 |
|  |  |  |  |  |  |  |
| Education (Years) | 16.7 (2.5) |  | 16.4 (2.9) |  | 0.90 | 0.37 |
|  |  |  |  |  |  |  |
| Smoker (% Yes) | 2.6% |  | 3.3% |  | 0.09 | 0.77 |
|  |  |  |  |  |  |  |
| Comorbidity Score | 2.75 (.97) |  | 2.92 (1.0) |  | 1.19 | 0.24 |
|  |  |  |  |  |  |  |
| Footnote: Comorbidity score is derived from the Charlson Comorbidity Index^74^ | | | | | | |
